# Cognitive Decline in Hospitalized Older Adults: A Scoping Review

**DOI:** 10.1111/psyg.70102

**Published:** 2025-10-10

**Authors:** Sara Escriche‐Martinez, Unai Diaz‐Orueta, Carlos Gala‐Serra, Esther Sierra‐Martínez, Ginesa López‐Crespo, Raúl López‐Antón

**Affiliations:** ^1^ Departamento de Psicología y Sociología Universidad de Zaragoza Teruel Spain; ^2^ Department of Psychology Maynooth University Maynooth Co. Kildare Ireland; ^3^ Hospital San José Teruel Spain; ^4^ Hospital San Juan de Dios Zaragoza Spain

**Keywords:** cognitive decline, hospitalization, inpatients, older adults, scoping review

## Abstract

Older adults often experience rapid cognitive decline following hospitalization, especially those with severe illness and extended stays. Despite known links between increasing patient age and cognitive decline, 30% of older adults without major pre‐existing conditions prior to medical admission show potential undiagnosed cognitive decline. This scoping review aims to map the prevalence, assessment methods, and associated factors of cognitive decline among hospitalized older adults. We conducted an exploratory review in accordance with the Joanna Briggs Institute (JBI) methodological framework and the PRISMA‐ScR guidelines. The review targeted studies published between January 2018 and March 2025 in English or Spanish that reported in‐hospital cognitive assessments in individuals aged 65 years and older. ‘Cognitive decline’ was operationally defined as performance below established cut‐off scores on validated tools, such as the Mini‐Mental State Examination (MMSE ≤ 23) or the Montreal Cognitive Assessment (MoCA < 26), administered during hospitalization. Databases consulted included PubMed, Web of Science, and ScienceDirect. A narrative synthesis was undertaken to organise findings by study design, cognitive instruments, prevalence rates, and associated factors. A total of thirty studies met the inclusion criteria. Most employed cross‐sectional or prospective cohort designs, with wide variability in hospital settings, timing of assessments, and cognitive tools used. The reported prevalence of cognitive impairment ranged from 10% to 85%, depending on the assessment tools and population characteristics. MMSE and MoCA were the most frequently used tools. Associated factors included advanced age, comorbidities, pre‐existing cognitive decline, and frailty. Methodological heterogeneity hindered meta‐analysis, but it also revealed important limitations in the comparability of the studies. This review identifies substantial heterogeneity in the assessment and reporting of cognitive decline among hospitalized older adults. The findings highlight the need for standardized screening protocols and improved methodological consistency to optimise the detection, cross‐study comparability, and clinical relevance of cognitive assessments in hospital settings.

## 1 Introduction

Although hospital admissions are common throughout life, they tend to become more frequent among those aged 65 and over [1, 2]. The higher likelihood of chronic diseases and an increased rate of complications during hospital stays lead to greater complexity in older adult patients, often resulting in longer hospitalizations and a more rapid cognitive decline during and after a hospital stay [3, 4, 5]. Cognitive decline typically refers to a measurable deterioration in one or more cognitive domains—such as attention, memory, or executive function—that exceeds expected age‐related changes and may indicate pathological processes or acute insults [6]. This decline can be transient or persistent, and in the context of hospitalization, may arise from acute illness, stress, or iatrogenic factors, potentially impacting functional recovery and long‐term outcomes. This cognitive deterioration is related to poorer outcomes, especially during acute medical admissions [7].

Despite this evidence linking hospital stays with cognitive decline, much of the existing research has focused on other related conditions such as delirium or frailty. In a recent systematic review [8], it showed a 34% prevalence of frailty and 21% of delirium in hospitalized older adults.

Studies have suggested that delirium contributes to long‐term cognitive decline, particularly in populations of vulnerable older adults [9, 10]. Frailty is another well‐known condition associated with poorer outcomes during hospitalizations. Studies have shown that frailty, when combined with cognitive decline, significantly raises the risk of hospitalization and functional decline [11].

The existing literature on neuropsychology and cognitive function has already demonstrated a relationship between hospitalizations in the older adult population and cognitive decline [4]. However, the association between hospitalization and decline in older individuals is often underestimated because, despite the well‐known relationship between increasing patient age and cognitive decline [12], 30% of advanced age individuals without major pre‐existing conditions prior to medical admission show potential undiagnosed cognitive decline [3]. Notwithstanding all these findings, there are few studies that focus specifically on cognitive decline in hospitalized older adults [3, 5, 7, 12]. In these emerging and methodologically diverse fields, exploratory reviews offer a valuable approach to comprehensively map the nature, scope, and extent of available evidence. While systematic reviews [13] have addressed long‐term cognitive outcomes after hospitalization, scoping reviews are better suited to examine how a topic has been researched, what types of evidence exist, and where significant knowledge gaps persist. In this context, the present scoping review focuses specifically on cognitive decline that occurs during hospitalization, incorporating a broader set of study designs and outcome measures. This exploratory and flexible approach, aligned with the Joanna Briggs Institute (JBI) methodology and PRISMA‐ScR guidelines, allows us to map conceptual and methodological variability and support future directions in clinical research.

## 2 Method

The proposed scoping review was conducted in accordance with the recommendations by JBI methodology for scoping reviews [14]. In addition, the search methods implemented in this scoping review were designed and executed in alignment with the Preferred Reporting Items for Systematic Reviews and Meta‐Analyses (PRISMA) criteria [15].

### 2.1 Search Strategy

As the purpose of this research is to investigate how existing studies have examined the association of hospitalizations with cognitive decline among older inpatients, conducting a scoping review was an appropriate methodology, as it allowed for the expansion of the research's scope. In this review, cognitive decline was defined as any reduction in cognitive performance identified during hospitalization using validated tools (e.g., Mini Mental State Examination (MMSE) ≤ 23, Montreal Cognitive Assessment (MoCA) < 26). Tools screening for delirium (e.g., Confusion Assessment Method—CAM) or both for delirium and cognitive performance (4AT—Assessment Test for Delirium and Cognitive Impairment) were also considered, as well as clinical criteria. Studies using the term cognitive impairment were included if the authors referred to acute or hospital‐related deterioration.

We independently searched three databases—PubMed, Web of Science, and ScienceDirect—from January 1st, 2018 to January 16th, 2024. The decision to restrict the search to PubMed, Web of Science, and ScienceDirect was based on their complementary strengths in indexing peer‐reviewed studies across clinical medicine, biomedical sciences, and interdisciplinary health research. Prior to launching the review, we conducted a pilot overlap analysis, which revealed substantial coverage of the core literature on in‐hospital cognitive decline across these databases. This triangulation allowed for efficient identification of relevant studies from both medical and psychological domains. Regarding the temporal restriction (i.e., from 2018), this decision was informed by the emergence of electronic health records (EHRs) and evolving diagnostic practices over the past decade, which have improved the consistency and documentation of in‐hospital cognitive assessments. By focusing on this recent time frame, we sought to ensure greater comparability among studies and capture the most up‐to‐date clinical and methodological developments.

We used the boolean operators “AND” and “OR” with the following search terms: “Length of stay” OR “Hospitalization” OR “Inpatients” OR “discharge” OR “Hospital admission” OR “Admitted Patients” AND (Cognit* OR Neuropsy*) AND (Function OR Disorders OR Impairment OR Decline OR Profile OR Phenotype OR Deficits OR Performance) AND (“aged”) NOT (“cancer*” OR “schiz*” OR “bipolar disorder”). We restricted this search to English or Spanish language. We performed a final, additional search including papers published since January 2024 to March 2025, but no additional studies identified in this period matched the scope of this review.

### 2.2 Study/Source of Evidence Selection

Following the search, all results were exported and uploaded into Rayyan Systems Inc. (www.Rayyan.ai), an online software for collaborative systematic reviews. A total of 980 results were identified and 815 eligible studies remained, following the removal of duplicate references. A three‐stage screening and selection process was conducted by two independent reviewers, blind to each other, with a third reviewer resolving disagreement.

This scoping review included articles that met the following inclusion criteria: focused on (1) older adults aged 65 years and over, (2) condition and progress of the patients along hospital stays, (3) all types of cognitive assessments conducted during hospital stays, including studies that employed the term *cognitive impairment* when referring explicitly to acute or hospital‐related cognitive deterioration, and (4) articles published in the period from 2018 to 2024 published in English or Spanish language. Titles and abstracts were screened to determine if they met inclusion criteria; those that did not were excluded from this review. Furthermore, articles that were review articles, meta‐analyses, commentaries, theses, case reports, or studies lacking direct engagement with hospitalized older adults were also excluded from this review.

### 2.3 Data Extraction

A total of 815 eligible studies remained following the extraction of duplicate references using Rayyan Systems Inc. (www.Rayyan.ai). Titles and abstracts were reviewed to determine eligibility. A significant number of studies were excluded based on the inclusion criteria for this study. The main reasons to exclude studies at this point were: wrong publication type, focus on prevalence of specific pathologies, COVID‐19 outcomes, specific medication treatments, outpatient population, psychiatric population, paediatric population, and test validation.

Following the screening process, a total of 256 studies were inspected further in full‐text review by two reviewers, using Rayyan Systems Inc. (www.Rayyan.ai) “*blind mode*” to ensure relevance to the review and overall credibility. This research panel examined full‐text versions to decide the inclusion or exclusion of each study. One of the reviewers was in charge of resolving any disagreements after finishing the review process.

Numerous references were focused on a different population, such as end‐stage diseases, after‐discharge population, brain injury population, organ transplantation, and patients with dementia. Several others were centred on mortality, including predictive factors, mortality risk after different surgical interventions, and long‐term survival after diverse pathologies. Various others directed attention to medical interventions or drugs, as in cardiovascular interventions, cardiac rehabilitation, physical training, anticholinergic drugs, benzodiazepine, or psychotropic drugs. A few other studies did not specify that cognition was assessed, despite being mentioned in the abstract or title. Finally, some of them were excluded because they were focused on other specific outcomes such as surgery, emergency hospitalization access or trends, physical activity, psychosocial problems, delirium, nutrition, neuroimaging or biomarkers, protocols, and trial descriptions.

**FIGURE 1:**
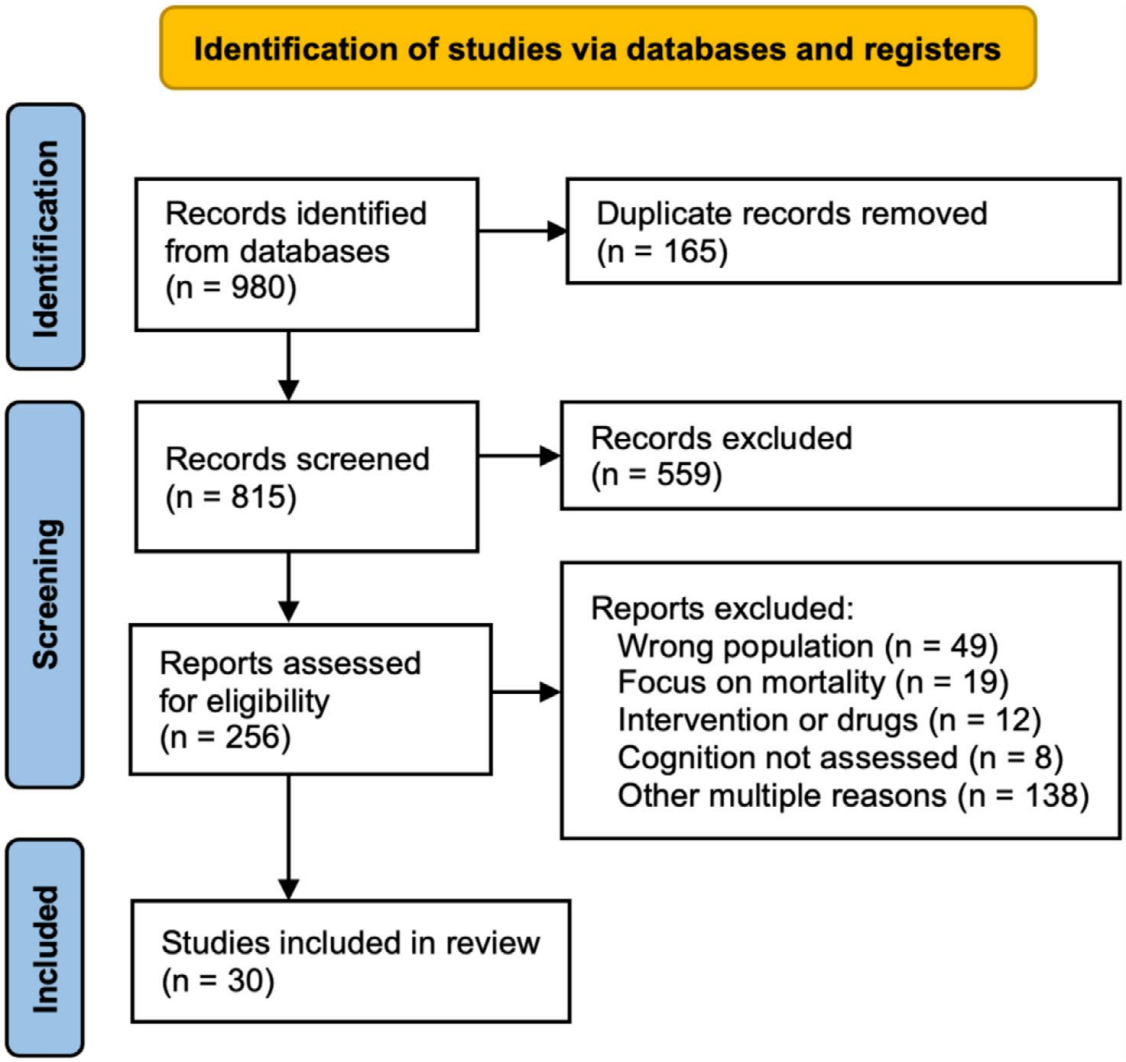
PRISMA flowchart for the literature search and article selection process (with two independent reviewers taking care of all stages, with the support of a third reviewer to solve disagreements or conflicts).

This procedure resulted in 30 eligible studies that were included in the scoping review. Two independent reviewers extracted key information from each selected study including study type (design), number and characteristics of the participants, measures and assessment techniques used, length of hospital stay, and the summary of main results. Figure 1 illustrates the flowchart detailing the literature search and article selection process. Table 1 summarises the main characteristics of the reviewed studies, Table 2 shows the prevalence data from these articles, Table 3 outlines the associated factors identified, and Table 4 presents the long‐term outcomes for each case.

**TABLE 1:** Study characteristics.

Authors and publication year	Country	Study design	Sample characteristics
Bertschi et al. [16]	Switzerland	Cross‐sectional study	*N* = 305 (65.6% women), median age 84 (79–89)
Durlach et al. [17]	Argentina	Prospective observational study	*N* = 270 (47.41% men), median age 74.5 (range 33–97)
Fernández‐Gonzalo et al. [18]	Spain	Prospective observational cohort study	*N* = 156; analysed sample *N* = 92 (38% women), age 64 (56–71)
Foran et al. [19]	Australia	Observational cohort study	*N* = 187 (community), *N* = 78 (inpatients), mean age 71.3 and 74.6. No gender distribution information was provided.
Hallgren et al. [20]	Sweden	Population‐based longitudinal study	*N* = 828 (40.7% men), mean age 63.4. Twin participants.
Hammami et al. [21]	Belgium	Cross‐sectional study	*N* = 124 (72.6% women), mean age 85.9 ± 5.5
Hou et al. [22]	China	Two‐stage cross‐sectional study	*N* = 1300, *N* = 1100 to develop model. Cognitive impairment group: median age 81 (75–85). Normal cognition group: median age 75 (68–81). Another 200 participants (aged 60–97 years) were included to assess the model.
Jiménez Mola et al. [23]	Spain	Cross‐sectional descriptive study	*N* = 557 (> 75 years), 74.7% women, mean age 86.66 (range 75–105)
Kamalzadeh et al. [24]	Iran	Cross‐sectional study	*N* = 205 (46.8% women), mean age 71.3 + 7.35
Karnatovskaia et al. [25]	USA	Prospective single‐center study	*N* = 299 (43.15% women), age range 50–75
Kindstedt et al. [26]	Sweden	Cross‐sectional study	*N* = 188 (63% women), mean age 84.2
Kunicki et al. [27]	USA	Prospective observational cohort study	*N* = 560 (58% women), mean age 76.7 ± 5.2
Lagarto et al. [28]	Portugal	Prospective observational study	*N* = 269 (100% men), mean age 81.0 ± 7.9
Mahanna‐Gabrielli et al. [29]	USA	Prospective observational cohort study	*N* = 167, robust: 64 (33.3%); prefrail: 72 (48.1%); frail: 31 patients (18.6%). No gender distribution information was provided. Age in robust: 70 (67, 74.5); prefrail: 70.5 (67, 75); frail: 71 (66, 74)
Mastaleru et al. [30]	Romania	Retrospective population‐based study	*N* = 663 (58.7% women), mean age 76.58 ± 6.56
Miao et al. [31]	China	Prospective observational cohort study	*N* = 2307 (36.4% women), mean age 64 ± 14. Age > 65 years, *n* (%) 1225 (53.1)
Mourao et al. [32]	Brazil	Cross‐sectional observational study	*N* = 50 (44% women), mean age 65.5 ± 11.7
Mudge et al. [33]	Australia	Prospective cross‐sectional study	*N* = 261 (60,65% men), mean age 76 (range 65–98)
Mutchie et al. [34]	USA	Retrospective cohort study (cross‐sectional analysis of baseline data from an observational cohort study)	*N* = 339 (50.4% women), mean age 81.6
Nagae et al. [35]	Japan	Prospective observational cohort study	*N* = 296 (57.3% women), mean age 84.7 ± 5.4
Niu et al. [36]	China	Cross‐sectional study	*N* = 192 (37% women), mean age 66.1 ± 10.6
Nomura et al. [37]	USA	Longitudinal observational study	*N* = 133, Non frail (*N* = 15) age 69.33 (7.90); Prefrail (*N* = 74) age 71.72 (6.93); Frail (*N* = 44) age 73.48 (8.09). No gender distribution information was provided.
Shami et al. [38]	USA	Secondary data analysis	*N* = 668 (57.6% women), median age 80 (range 65–85+)
Sprung et al. [39]	USA	Population‐based longitudinal study. Observational study.	*N* = 4587 (51% men), No hospitalization (*N* = 2965) Yes hospitalization (1 or More) (*N* = 1622), mean age 74 (range 69–81)
Sumida et al. [40]	Japan	Longitudinal observational study	*N* = 111 (50% men), median age 77 (range 71–85)
Tolley et al. [41]	Australia	Prospective, longitudinal, observational cohort study	*N* = 1716 (56.4% women), median age 83.4 (range 77.7–88.6)
Wiegand et al. [42]	Switzerland	Cross‐sectional and prospective analysis	*N* = 273 (38.1% men), mean age 79.4 ± 6.5
Wilke et al. [43]	Germany	Prospective observational chart review study	*N* = 30 (50% women), mean age 82.3 ± 6.6 (range 66–92)
Yamamoto et al. [44]	Japan	Cross‐sectional observational study	*N* = 885, mean age 78.9. No gender distribution information was provided.
Zipprich et al. [45]	Germany	Prospective observational study	*N* = 591 (Full delirium: *N* = 64, age 78.6 (8.5); Two positive items on the Confusion Assessment Test: *N* = 75, age 72.9 (8.1); No delirium: *N* = 452, age 77.7 (7.1). No gender distribution information was provided).

**TABLE 2:** Prevalence data.

Authors and publication year	Assessment instrument(s)	Cut‐off used	Assessment time point	Reported prevalence
Bertschi et al. [16]	MMSE, the timed up and go test (TUG), the nutritional risk screening (NRS 2002) and Functional Independent Measure (FIM)	No cut‐off (FIM quartiles used due to lack of validated thresholds)	Hospital admission	Sarcopenia prevalence: 22.6%; associated with lower FIM total and cognitive items (e.g., comprehension, social interaction) (no cognitive decline prevalence reported)
Durlach et al. [17]	Confusion Assessment Method for the Intensive Care Unit (CAM‐ICU), Basic Activities of Daily Living (BADL), Instrumental Activities of Daily Living (IADL), APACHE II and Charlson.	Delirium: CAM‐ICU criteria 1 + 2 plus 3 or 4; SSD (subsyndromatic delirium): ≥ 1 criterion altered without full criteria	Twice daily during ICU stay; repeated if symptoms of delirium appeared	Only SSD: 17.03%; Delirium or both: 22.96%
Fernández‐Gonzalo et al. [18]	Short‐IQCODE The National Adult Reading Test (NART)—Spanish version‐Subtest of Digits from the Wechsler Adult Intelligence Scale version III (WAIS III) Subtest of Spatial Span from the Wechsler Memory Scale version III (WMS III) Rey Auditory Verbal Learning test Benton Visual Retention test Subtest of Symbol Search (WAIS III) Stroop Colour and Word test Trail Making Test FAS	Short‐IQCODE > 3.56 (used to exclude preexisting cognitive impairment)	Preexisting cognitive impairment was assessed at ICU admission (daily screening by a critical care nurse). A complete and comprehensive neuropsychological assessment was administered 1 month after ICU discharge.	Global cognitive impairment (classical approach): 47%; domain‐specific deficits: speed of processing 36%, executive function 31%, memory retrieval 25%, memory storage 22%.
Foran et al. [19]	MMSE, Frontal Assessment Battery (FAB), QuickSort; DASS‐21	MMSE < 24; FAB < 11 (non‐impaired: MMSE ≥ 25; FAB ≥ 12)	During hospitalization (inpatient; test–retest while in hospital (*n* = 46))	Inpatient sample: MMSE impaired (< 24): 31/78 (40%); FAB impaired (< 11): 23/78 (29%); MMSE or FAB (or both) impaired: 39/78 (50%).
Hallgren et al. [20]	MMSE score; Depressive symptoms, as measured by the Center for Epidemiologic Studies Depression Scale (CES‐D); Self‐rated health scale: individuals' current general health, current health versus health 5 years ago, own health compared with others' health and limitations in activities due to health. Total number of hospitalizations was extracted from the NPR (from the Swedish National Patient Register) SATSA cognitive test battery assessed four cognitive domains, verbal, spatial/fluid, memory and processing speed abilities and a global cognitive composite score. Verbal: Information Subtest (from [WAIS‐R] (Wechsler, 19 819, Synonyms, and Analogies)). Spatial/fluid: Figure Logic, Block design (WAIS‐R), and Card Rotation. Memory: using Digit Span (WAIS‐R) and Thurstone's Picture Memory Task. Processing speed abilities: Symbol Digit (an inverted version of the Symbol Digit Substitution task (Smith, 1982), and Figure Identification).	MMSE < 25 or ≥ 10% decline from previous IPT triggered dementia evaluation; diagnosis based on DSM criteria	In‐person testing approximately every 3 years, up to 25 years, to evaluate cognitive change before and after hospitalization.	Not reported (latent growth curve modelling reports change estimates, not % decline)
Hammami et al. [21]	Cognitive, nutritional status and physical activity were assessed using Mini Mental State Examination score (MMSE), Mini Nutritional Assessment score (MNA), and Katz score, respectively. Frailty syndrome was evaluated using the modified Short Emergency Geriatric Assessment (SEGA) score. Medication and medical history were recorded. Analysed biochemical parameters included C‐reactive protein (CRP), complete blood count, blood creatinine, vitamin D level, and serum protein electrophoresis	MMSE < 22; MNA < 17; GDS‐15 > 4	During hospital stay (data collected between January and March 2018)	Dementia: 39.5%; Confusion: 48%; Malnutrition (MNA < 17): 50.4%; ADL dependency (Katz < 6): 84.6%; Polypharmacy (≥ 5 drugs): 71%; Depression (GDS‐15 > 4): 21%
Hou et al. [22]	Cognitive status was assessed using the Mini‐Mental State Examination (MMSE) scale and clinical diagnoses. Depression was diagnosed with clinical symptoms and the geriatric depression scale (GDS). Central obesity was diagnosed based a waist‐hip ratio (WHP)	MMSE ≤ 26 and clinical judgement; GDS > 10 and clinical symptoms; WHP ≥ 0.9 for men and 0.85 for women.	During hospital stay (cross‐sectional data from January 2015–December 2020)	Cognitive impairment: 57.5%; dementia: 4.9%; mild CI: 36.0%; moderate CI: 16.4%; severe CI: 5.2%
Jiménez Mola et al. [23]	Barthel Index to evaluate activities of daily living Orthogeriatric medical diagnosis according to DSM‐V criteria	No numerical cut‐off; cognitive impairment levels categorized into severe/moderate, mild, and no impairment based on DSM‐V criteria.	From hospitalization to hospital discharge (data extracted from medical records)	No impairment: 54.9% (*n* = 293); mild: 20.4% (*n* = 109); moderate/severe: 24.7% (*n* = 132)
Kamalzadeh et al. [24]	Mini‐Mental State Examination (MMSE), Mini‐Cog test, Geriatric Depression Scale (GDS‐15), Activities of Daily Living–Instrumental Activities of Daily Living (ADL‐IADL) scale, and socioeconomic questionnaires	MMSE < 26/30 or Mini‐Cog < 3, followed by clinical evaluation using DSM‐5 criteria for dementia/major neurocognitive disorder.	During hospital admission (from October 2017—March 2018)	Dementia: 22%.
Karnatovskaia et al. [25]	Montreal Cognitive Assessment‐Blind (MoCA‐blind); Hospital Anxiety and Depression Scale (HADS‐A and HADS‐D); Impact of Events Scale‐Revised (IES‐R)	MoCA‐blind: < 18; HADS‐A/HADS‐D: ≥ 8; IES‐R subscore: ≥ 1.6	Within 96 h after ICU discharge, while still hospitalized	MoCA‐blind < 18: 58%; HADS‐D ≥ 8: 37%; HADS‐A ≥ 8: 45%; IES‐R subscore ≥ 1.6: 39%; Cognitive impairment at 3 months: 28%
Kindstedt et al. [26]	Assessment Tool for Hospital Admissions Related to Medications 10 (AT‐HARM10 to classify possibly medication‐related admissions (MRAs)); 4‐item version of the Gottfries' cognitive scale for cognitive screening	No more than one negative answer on the 4‐item Gottfries' scale to indicate cognitive impairment (sensitivity 97.8%, specificity 92.5%). In analysis, lowest possible score (0/4 correct) was compared against all other scores combined.	During ward stay	36% of hospital admissions classified as possibly medication‐related. Distribution of MRAs by cognitive test score: 0/4 = 15%, 1/4 = 32%, 2/4 = 47%, 3/4 = 42%, 4/4 = 34%.
Kunicki et al. [27]	Screening for dementia included medical record review, patient report, Modified Mini‐Mental State (3MS). Comprehensive battery of 11 neuropsychological tests was assessed preoperatively and across multiple points postoperatively to 72 months of follow‐up (including tests of attention, memory, language, and executive function). Delirium was assessed daily during hospitalization using the Confusion Assessment Method (CAM). Charlson comorbidity scale score, surgery type (orthopaedic, vascular, or gastrointestinal), and impairments in basic and instrumental activities of daily living (IADLs). 15‐item Geriatric Depression Scale (GDS) Proxy‐rated Informant Questionnaire on Cognitive Decline in the Elderly (IQCODE)	No cut‐off for impairment; GCP analysed as a continuous score, scaled with a mean of 50 (SD 10) based on a nationally representative sample of adults ≥ 70 years, and corrected for retest effects using nonsurgical comparison sample. Delirium classification if either CAM or medical record criteria were met on any hospital day.	Baseline assessment and medical record screening were conducted within 30 days before surgery (median, 9 days; IQR, 12 days). After discharge, follow‐up assessments occurred at 1, 2, 6, 12, 18, 24, 30, 36, 48, 60, and 72 months.	Post‐operative delirium: 24% (*n* = 134)
Lagarto et al. [28]	Level of arousal: Richmond Agitation and Sedation Scale (RASS). Delirium assessment: Confusion Assessment Method (CAM) and DSM‐5 criteria. Cognitive assessment: Mini‐Mental State Examination (MMSE). Dementia assessment: Informant Questionnaire on Cognitive Decline in the Elderly (IQCODE‐SF). Dementia severity: Global Deterioration Scale. Sociodemographic data and comorbidities: Collected from clinical files, including the Charlson Co‐Morbidity scale and Barthel Index	IQCODE‐SF score ≥ 3.9 for dementia; RASS ≤ −3 considered too sedated for cognitive assessment; positive CAM screening confirmed with DSM‐5 criteria for delirium.	Within the first 72 h of admission and every other day until discharge	Baseline delirium prevalence 15.4%; cumulative prevalence 23.4% (40.8% in patients with dementia vs. 12% without dementia)
Mahanna‐Gabrielli et al. [29]	Preoperative frailty was determined using the FRAIL scale, a simple questionnaire that categorises patients as robust, prefrail, or frail. Delirium was assessed with the Confusion Assessment Method for the intensive care unit (CAM‐ICU). Neuropsychological battery (California Verbal Learning Test II and Uniform Dataset Battery (UDS):Trail Making Test, subtests from the Wechsler Adult Intelligence Scale, Logical Memory Story A, Immediate and Delayed Recall, Animal and Vegetable verbal fluency, Boston Naming Test, and the Mini‐Mental Status Examination)	CAM‐ICU criteria for delirium (positive if criteria I and II plus either III or IV met); POCD defined as ≥ 1 SD decline in cognitive score from baseline to 3 months.	Delirium was assessed (CAM‐ICU) twice daily, starting in the recovery room until hospital discharge. Neuropsychological battery performed preoperatively and at 3 months afterward.	Postoperative cognitive dysfunction (POCD): 12.3%; delirium: 22.1%.
Mastaleru et al. [30]	Cognitive status was assessed using the Mini‐Mental State Examination (MMSE), the degree of dependence/independence in ADL and IADL, depression assessed using the 15‐item Geriatric Depression Scale (GDS), nutritional status using the Mini Nutritional Assessment (MNA). Frailty status was determined using the Fried criteria. The patients were assessed for the presence of the following criteria: weakness, weight loss, decreased muscle strength, decreased physical activity, decreased walking speed	Fried criteria: 0 criteria = non‐frail, 1–2 criteria = pre‐frail, ≥ 3 criteria = frail.	During hospital admission, based on retrospective review of medical records from a 13‐month period.	Moderate cognitive dysfunction: 23.3% Frailty: 73% Moderate depression: 45.1% Independent in ADLs: 64.2% Moderately dependent in IADLs: 49.8%
Miao et al. [31]	The Mini‐Cog test (two components that include a three‐item recall and a clock drawing) Depressive symptoms were evaluated using the Patient Health Questionnaire‐2 (PHQ‐2). Quality of life status was measured using the Kansas City Cardiomyopathy Questionnaire‐12 (KCCQ‐12)	Mini‐Cog ≤ 2 for cognitive impairment	Data were collected during the index hospitalization and followed up at 1, 6, and 12 months post‐discharge.	Normal cognition before discharge: 71.9% (*n* = 1658); new‐onset cognitive impairment at 1 month: 13.8% (*n* = 229). Cognitive impairment before discharge: 28.1% (*n* = 649); recovered to normal cognition at 1 month: 48.5% (*n* = 315).
Mourao et al. [32]	Trial of Org 10 172 in Acute Stroke Treatment (TOAST). The Oxfordshire Community Stroke Project (OCSP) was used to classify the clinical characteristics according to the anatomical location of the lesion. The NIHSS4 was used to quantify neurological impairment. The Mini‐Mental State Examination (MMSE) and Frontal Assessment Battery Assessment (FAB) were used to assess cognition. The Alberta Stroke Program Early Computed Tomography (CT) Score (ASPECTS) was applied to detect and quantify brain tissue changes by means of early ischemic signs in the territory of the middle cerebral artery, the most often affected area in stroke. The examination of dysphagia was clinically performed using the Gugging Swallowing Screen (GUSS) and Functional Oral Intake Scale (FOIS)	ASPECTS = 0 (diffuse ischemia in the whole territory of the middle cerebral artery) Functional Oral Intake Scale (FOIS): FOIS gradation GUSS: 20 = normal, 15–19 = mild dysphagia, 10–14 = moderate dysphagia, 0–9 = severe dysphagia; MMSE/FAB cut‐off according to education level	At hospital admission (≤ 24 h after stroke), 72 h after hospitalization, and at hospital discharge.	Dysphagia at admission: 50% Hypertension: 67.3% Anterior ischemia: 34% Previous history of stroke: 50%
Mudge et al. [33]	4AT and Carer Questionnaire. ADL was recorded from daily nursing documentation	Likely cognitive impairment without delirium: 4AT 1–3 Cognitive impairment with probable delirium: 4AT > 3	Audit day: 14 March 2018	Cognitive impairment: 43% Probable delirium: 24%
Mutchie et al. [34]	There was no measure of cognitive function pre‐fracture or during their hospitalization. Cognitive testing within 22 days of admission. Modified Mini‐Mental State Examination (3MS). Hospital Record. Modified Charlson Comorbidity Index (CCI)	3MS ≤ 78 for cognitive impairment	No measure during hospitalization, testing within 22 days of admission.	Source of Cognitive Impairment Identification (SCI): 12.7% ‘3MS Only’ (*n* = 42), 11.5% ‘Hospital Record Only’ (*n* = 38), 9.4% ‘Both’ (*n* = 31), and remaining participants ‘No CI’ (*n* = 219).
Nagae et al. [35]	The five domains of Intrinsic Capacity (IC) proposed by the WHO were evaluated: locomotion, cognition, vitality, sensory, and psychological capacity. Locomotion: mobility category of the Barthel index (BI) Cognition was assessed using the Mini‐Mental State Examination (MMSE). Vitality was assessed by the Mini‐Nutritional Assessment‐Short Form (MNA‐SF). Sensory capacity was based on examination for visual and hearing impairment (yes/no) by a physician. Psychological capacity was assessed using the Geriatric Depression Scale‐15 (GDS‐15). Comorbidities were evaluated using the Charlson Comorbidity Index	MMSE scores of 0–9,10–26, and 27–30 were assigned IC (cognition) scores of 0–2 (0 = severely impaired, 1 = partially impaired, 2 = slightly impaired or preserved) GDS‐15 scores of 0–4, 5–9, and 10–15 were assigned IC (psychological) scores of 2, 1, and 0, respectively.	At hospital admission (within 48 h of admission)	95.6% of participants had impairment in at least one IC domain.
Niu et al. [36]	Beijing version of the Montreal Cognitive Assessment; Chinese version of the Zung Self‐Rating Depression Scale (SDS)	MoCA < 26 for cognitive impairment. SDS ≥ 53 for depression	Within 24 h before discharge from the hospital	Cognitive impairment: 53.6% Hyperuricemia: 54.7%
Nomura et al. [37]	The tests assessed a number of cognitive domains known to be affected by cardiac surgery. The test battery consisted of the Rey Auditory Verbal Learning Test, Rey Complex Figure Test, Controlled Oral Word Association Test, Symbol Digits Modalities Test, Trail Making Tests A and B, and Grooved Pegboard Test Fried frailty scale Confusion Assessment Method (MMSE, digit span forward/backward, and timed months of the year backward) Richmond Agitation Sedation Scale	Mini‐Mental State Examination12 < 23 (exclusion criteria) Composite cognitive Z‐score from baseline; decline calculated as the difference in scores between baseline, 1 month, and 1 year post‐surgery Nonfrail = 0; Prefrail = 1–2; Frail = 3–5 RASS: −4 or − 5 (coma)	Neuropsychological testing was generally performed within 2 weeks of surgery and then 4–6 weeks and 1 year after surgery.	Nonfrail: 13% delirium; Prefrail: 48% delirium; Frail: 48% delirium
Shami et al. [38]	Mini‐Cog (three‐item recall test and clock drawing) and Reported Edmonton Frailty scale (REFS)	Mini‐Cog ≤ 2 for cognitive impairment. REFS (excellent = 0–1, fair = 2–4, poor = 5–8)	Within 24 h of admission	Cognitive impairment: 35%
Sprung et al. [39]	APOE genotyping. Short Test of Mental Status, a medical history review, and neurologic examination	No cut‐off; continuous z‐scores used	Baseline and 15‐month intervals	35.4% (1622 of 4587 participants) had ≥ 1 hospitalization during follow‐up
Sumida et al. [40]	Functional Independence Measure (FIM) (FIM‐Cognitive and FIM‐Physical); Mini‐Nutrition Assessment‐Short Form (MNA‐SF)	FIM‐Cognitive score ≤ 32 (impaired cognition) FIM‐Cognitive score 5 (full assistance)	Admission and discharge	50.5% (56 of 111 patients) classified as impaired cognition
Tolley et al. [41]	Comprehensive Geriatric Assessment (CGA): demographic, medical, nutritional, functional, and psychological assessments. Clinical Frailty Scale (CFS) Comorbidity Index (CCI), short Confusion, Assessment Method (short CAM), Cumulative Illness Rating Scale (CIRS), Mini‐Mental State Examination (sMMSE), Montreal Cognitive Assessment (MoCA) and Rowland Universal Dementia Assessment Scale (RUDAS), Hospital Anxiety and Depression Scale (HADS)	sMMSE < 24 MoCA < 26 RUDAS < 23 Dementia diagnosis in medical records CFS score (1–9): 8–9 Very severely frail/terminally ill	CGA within 48 h after admission and before discharge	Cognitive impairment: 67% (1149 of 1716 participants) Delirium: 25.8% (448 of 1716 participants)
Wiegand et al. [42]	Geriatric Depression Scale (GDS‐15); Mini‐Mental State Examination (MMSE); Mini Nutritional Assessment (MNA); adapted version of Fried frailty status; Grip strength measurement; Physical activity The assessments were conducted by trained interviewers within the first four days of hospital admission	MMSE < 17 (17–23) GDS‐15 ≥ 5 (possibly depressed) Mini Nutritional Assessment (MNA) < 17 (malnourished) adapted version of Fried frailty status: pre‐frail if they had 1 or more points.	Within the first four days of hospital admission	Cognitively normal 55% (MMSE ≥ 27) Depressive symptoms: 19%
Wilke et al. [43]	Mini‐Mental‐Status‐Examination (MMSE), Geriatric Depression Scale (GDS), Delirium Rating Scale Revised 98 (DRS‐R‐98), Laboratory parameters—CRP and WBC, Barthel Index (BI), Brain imaging biomarkers	MMSE ≤ 24 (cognitive impairment) DRS‐R‐98 ≥ 17.75 (presence of delirium)	Baseline examination and follow‐up examinations (MMSE + DRS‐R‐98) planned in a 2–3‐day rhythm.	Prediagnosed dementia 36.7% Prediagnosed Mild Cognitive Impairment 20% Prediagnosed depression 43.3%
Yamamoto et al. [44]	Mini–mental state examination and STMT‐R (revised simplified short‐term memory recall test)	STMT‐*R* ≤ 4 (cognitive dysfunction)	Within a week of admission	Cognitive dysfunction 63,32% (460 of 726 patients)
Zipprich et al. [45]	Richmond Agitation‐Sedation Scale (RASS); Montreal Cognitive Assessment (MoCA); Confusion Assessment Test (CAM) or Glasgow Coma Scale and CAM‐ICU; If MOCA < 26, then Informant Questionnaire on Cognitive Decline in the Elderly (IQCODE); Identification of Seniors at Risk (ISAR) questionnaire; Study of Osteoporotic Fractures (SOF) frailty index	MoCA < 26 points (cognitive deficits)	The point prevalence and risk factors of delirium were assessed during the patients' stay. Outcome scores were assessed after 6, 12, 18, and 36 months.	Delirium 10.8% Mild cognitive impairment 33.3% MoCA < 21 39.8%

**TABLE 3:** Associated Factors.

Authors and publication year	OR/RR	Covariates
Bertschi et al. [16]	OR = 1.28 (95% CI 1.02–1.59) for comprehension; OR = 1.27 (95% CI 1.03–1.57) for social interaction	Not reported
Durlach et al. [17]	Delirium (multivariate): MVS (OR 20.82; 95% CI 3.47–124.75) Cognitive impairment (OR 7.84; 95% CI 2.57–23.89) Age ≥ 75 years (OR 3.02; 95% CI 1.19–7.64) Neurological reason for admission (OR 8.93; 95% CI 2.14–37.28) Skin sores (OR 48.11; 95% CI 2.54–907.98) Polypharmacy during admission (OR 4.57; 95% CI 1.31–15.94) Digestive rest (OR 3.04; 95% CI 1.12–8.19) Shock as reason for admission (OR 4.94; 95% CI 1.05–23.22) SSD (multivariate): IADL < 8 (OR 4.14; 95% CI 1.85–9.26) Enteral nutrition (OR 26.05; 95% CI 4.34–156.35) Illiterate or primary education (OR 2.83; 95% CI 1.23–6.48) Reason for admission other than cardiovascular (OR 4.04; 95% CI 1.26–12.91) Charlson > 5 (OR 2.46; 95% CI 1.10–5.52)	Multivariate logistic regression model including listed predictors.
Fernández‐Gonzalo et al. [18]	Age (OR 1.05; 95% CI 1.00–1.01) Gender (OR 2.81; 95% CI 1.01–7.84) Cognitive reserve (OR 0.37; 95% CI 0.16–0.83) Days with opioids (OR 0.17; 95% CI 0.03–1.08; trend)	Final multivariable model including age, gender, cognitive reserve, and days with opioids
Foran et al. [19]	Positive likelihood ratios (LR+) for QuickSort predicting impairment (MMSE or FAB or both): cut‐score < 4 → sensitivity 0.41, specificity 0.94, LR+ 6.95 (95% CI 3.75–12.86); cut‐score < 10 → sensitivity 0.88, specificity 0.77, LR+ 3.75 (95% CI 2.81–5.00). Note: these are diagnostic accuracy metrics, not odds ratios or relative risks.	Not reported (diagnostic accuracy analysis only)
Hallgren et al. [20]	Not reported (latent growth curve models used; no OR/RR calculated)	Main model: age, sex, education. Additional models: number of illnesses, self‐rated health, depression, number of admissions (hospitalized participants), years between first hospitalization and subsequent IPT, dementia.
Hammami et al. [21]	Dementia (OR 4.04; 95% CI 1.1–4.7), polypharmacy ≥ 5 (OR 3.2; 95% CI 1.01–6.7), living in nursing home (OR 3.42; 95% CI 1.5–4.9), Katz score < 6 (OR 4.7; 95% CI 1.9–5.6).	Dementia, polypharmacy ≥ 5, living in nursing home, Katz score < 6, age, sex, depression, MNA score, CRP, albumin, confusion.
Hou et al. [22]	Age: OR 1.091 (95% CI 1.072–1.110); Primary school or below: OR 1.412 (95% CI 1.087–1.833); Diabetes: OR 1.373 (95% CI 1.035–1.822); Depression: OR 1.910 (95% CI 1.317–2.770)	Age, sex, educational level, BMI, central obesity, smoking, drinking, diabetes, hypertension, depression, hypercholesterolemia.
Jiménez Mola et al. [23]	Not applicable (no OR/RR reported)	Not applicable (no multivariable model reported)
Kamalzadeh et al. [24]	Gender (OR 1.98); Age ≥ 71 years (OR 1.96); History of unemployment (OR 1.79); Number of children ≥ 4 (OR 2.53).	Gender, age, educational level, marital status, occupation, income level, number of children, nicotine use, other substance use, sedative‐hypnotic use, ever been to a psychiatrist/psychologist, history of head trauma, stroke, Parkinson's disease, hypertension, diabetes mellitus, ischemic heart disease, depression, other psychiatric disorders, hyperlipidemia, cancer, hearing impairment, visual impairment, falls history, ADL dependency, IADL dependency, GDS score, polypharmacy.
Karnatovskaia et al. [25]	HADS‐D ≥ 8: Depression OR 4.62 (1.63–13.11), Drug abuse OR 23.81 (3.46–163.93), PTSD OR 17.67 (3.07–101.85), Invasive ventilation days OR 1.68 (1.01–2.81), PT in ICU OR 0.28 (0.11–0.69). HADS‐A ≥ 8: Anxiety OR 3.69 (1.31–10.37). MoCA‐Blind < 18: In‐hospital MoCA‐Blind OR 0.60 (0.48–0.75), Invasive ventilation days OR 0.54 (0.32–0.89), NIV OR 0.26 (0.08–0.80), Neuromuscular block OR 0.21 (0.06–0.80). IES‐*R* ≥ 1.6: Age OR 0.92 (0.85–0.98), Anxiety OR 3.69 (1.31–10.37), NIV OR 3.25 (1.04–10.15), Neuromuscular block OR 0.18 (0.05–0.61).	Age, sex, ICU type, APACHE III, Charlson index, ICU LOS, delirium, benzodiazepines, steroids, propofol, invasive ventilation, non‐invasive ventilation, neuromuscular block, physical therapy, anxiety, depression, PTSD, drug abuse, alcohol abuse, baseline psychocognitive scores.
Kindstedt et al. [26]	Lowest cognitive test score (0/4) vs. all other scores: OR 0.28 (95% CI 0.10–0.84). Gottfries' 4‐item cognitive test score—0 positive answers vs. 1–4 positive answers: OR 0.31 (95% CI 0.10–0.93; *p* = 0.037).	Gottfries' 4‐item test score (0 positive answers), Cohabitant status, home health care
Kunicki et al. [27]	Not applicable (cognitive trajectories modelled as adjusted mean differences in GCP over time by delirium status)	Age, sex, race, preoperative IQCODE score, any IADL impairment at baseline, Geriatric Depression Scale score, Charlson Comorbidity Index score, surgery type
Lagarto et al. [28]	Dementia: independently associated with moderate/severe sedation (RASS ≤ −3) at admission (*p* < 0.001). Greater dementia severity (GDS) predicted lower RASS scores in patients with RASS < 0 (*p* = 0.012). Presence of RASS ≤ −3 at admission and/or during hospitalization associated with increased mortality until discharge (HR: 6.1; 95% CI: 2.0–18.5; *p* = 0.001).	Dementia diagnosis; age; use of benzodiazepines, antipsychotics, antidepressants; dementia severity (GDS); comorbidity burden
Mahanna‐Gabrielli et al. [29]	Prefrail or frail vs. robust: OR = 2.7 (97.5% CI: 1.0–7.3); Baseline cognitive score: OR = 0.4 (97.5% CI: 0.2–0.7).	Age, sex, education, surgical duration, surgical type, American Society of Anesthesiologists physical status, frail or prefrail, robust, baseline cognitive score
Mastaleru et al. [30]	No Odds Ratio (OR) or Relative Risk (RR) values reported; correlations with frailty status (MMSE: r = −0.094, *p* = 0.039; MNA: r = −0.151, *p* = 0.001; GDS: *r* = 0.093, *p* = 0.046)	MMSE, MNA, GDS, ADL, IADL, chronic medication use
Miao et al. [31]	HR 1.35 (1.07–1.70) for cardiovascular death or HF rehospitalization in patients with new‐onset cognitive impairment. HR 1.17 (0.95–1.44) for persistent cognitive impairment (not statistically significant). HR 0.91 (0.73–1.13) for transient cognitive impairment (not statistically significant). HR 1.22 (1.00–1.49) for all‐cause death or rehospitalization in new‐onset cognitive impairment (borderline significance). HR 1.17 (0.99–1.40) for persistent cognitive impairment (not statistically significant). OR 1.21 (1.12–1.31) per 5‐year age increase; OR 1.67 (1.19–2.34) female sex; OR 1.45 (1.02–2.07) less than high school education; OR 1.56 (1.07–2.27) prior atherosclerotic CVDs; OR 1.08 (1.01–1.16) per 10‐point KCCQ‐12 decrease; OR 1.46 (1.22–1.74) per 1‐point Mini‐Cog decrease.	Age, sex, education level, marital status, NYHA functional class, new‐onset HF or acute decompensated HF, hypertension, diabetes, prior myocardial infarction, atrial fibrillation, stroke, peripheral artery disease, chronic obstructive pulmonary disease, systolic blood pressure, LVEF group, estimated glomerular filtration rate, NT‐proBNP tertiles, depressive symptoms, and self‐reported use of medications at 1 month (including renin–angiotensin system inhibitors, *β*‐blockers, aldosterone receptor antagonists).
Mourao et al. [32]	Not reported	Cognitive function (MMSE, FAB), neurological function (NIHSS ASPECTS), dysphagia severity (GUSS, FOIS)
Mudge et al. [33]	Not applicable	Not applicable (analyses compared 4AT cognitive impairment categories by age, sex, admission source, ward type, length of stay, and discharge destination)
Mutchie et al. [34]	Males had greater odds of CI identified by ‘Both’ (OR = 2.33, 95% CI [1.07, 5.10]) and ‘3MS Only’ (OR = 2.31, 95% CI [1.16, 4.58]) compared to females, but not ‘Hospital Record Only’ (OR = 1.42, 95% CI [0.71, 2.84]). After adjustment for age, education, and CCI, only the ‘Both’ category remained significant (OR = 2.70, 95% CI [1.15, 6.34]).	Age, education, Charlson Comorbidities Index (CCI); Source of Cognitive Impairment Identification (SCI); Charlson Comorbidities Index (CCI); Modified Mini‐Mental State Examination (3MS).
Nagae et al. [35]	In‐hospital death: IC composite score OR = 0.59 (95% CI 0.37–0.94); High IC vs. Low IC OR = 0.14 (0.02–1.13) Hospital‐associated complications (HACs): IC composite score OR = 0.71 (0.59–0.84); Cognition OR = 0.87 (0.83–0.92); Psychological OR = 0.51 (0.35–0.74)	Age, sex, education years, comorbidities, and medication numbers. HACs, Hospital‐Associated Complications. IC, Intrinsic Capacity.
Niu et al. [36]	Not applicable (standardized *β* coefficients from multiple linear regression are reported) *β* = −0.353 (age), *β* = 0.297 (education), *β* = −0.188 (hs‐CRP), *β* = −0.261 (SDS), *β* = −0.130 (serum UA, ΔR^2^ = 0.014)	Not applicable (hierarchical regression: age, gender, educational level, NYHA function class, LVEF, BMI, haemoglobin, hs‐CRP, eGFR, hypertension, type 2 diabetes, atrial fibrillation, SDS score, serum UA)
Nomura et al. [37]	Delirium incidence—Prefrail vs. Nonfrail: OR 6.43 (95% CI 1.31–31.64, *p* = 0.022); Frail vs. Nonfrail: OR 6.31 (95% CI 1.18–33.74, *p* = 0.031)	Age, gender, education, and logistic European System for Cardiac Operative Risk Evaluation
Shami et al. [38]	30‐day readmission: OR = 1.02 (95% CI: 0.64–1.64) 60‐day readmission: OR = 1.18 (95% CI: 0.79–1.75) 90‐day readmission: OR = 1.39 (95% CI: 0.94–2.06) 1‐year survival: OR = 0.82 (95% CI: 0.53–1.27)	Demographics, principal diagnosis, admission from home, functional capacity, self‐reported general health, comorbidities
Sprung et al. [39]	Not applicable (estimates of change in cognitive z‐score slopes reported)	Age, sex, education, marital status, education, smoking status, APOE ε4 genotype, cardiometabolic conditions, global cognitive z‐score.
Sumida et al. [40]	Age: OR 1.06 (95% CI 1.00–1.13); MNA‐SF: OR 0.73 (95% CI 0.56–0.95); FIM‐Physical score: OR 0.94 (95% CI 0.90–0.99)	Factors included in the multiple logistic regression model: Age, gender, smoking, AF, BMI, HR, SBP, DBP, Haemoglobin, ln(BNP), ln(HbA1c), ln(hs‐CRP), ln(Creatinine), Uric acid, Sodium, Potasium, MNA‐SF, LVEF, FIM‐Physical score, Anti‐dementia medication
Tolley et al. [41]	Improvement vs. no change: CIRS score OR = 1.044 (95% CI 1.016–1.072), Medications OR = 1.045 (95% CI 1.007–1.085) Worsening vs. no change: CIRS score OR = 0.954 (95% CI 0.922–0.988)	Variables included in final backward stepwise prediction model: cardiac admission, cognitive impairment, delirium, CCI score, CIRS score, medication, SPPB score, HADS anxiety score
Wiegand et al. [42]	OR = 2.62 (95% CI = 1.08–6.34) for discharge to acute geriatric care vs. home in depressed patients	Age, gender, study period, nutritional status, cognitive function (MMSE ≤ 17, 18–23, ≥ 24), grip strength (quartiles), frailty status (frail, pre‐frail, robust), comorbidities, presence of fractures (multiple vs. single)
Wilke et al. [43]	Regression estimates (*β*): −1.42 (95% CI: −1.90 to −0.92) decrease in DRS‐R‐98 total score per day; +0.93/day improvement in MMSE for male sex × time (baseline ≈ −4.45 points); +0.81/day improvement in MMSE for prediagnosed dementia × time (baseline ≈ −4.65 points); motor retardation, orientation, and visuospatial ability were negative predictors of MMSE final scores	Final LME model: DRS‐R‐98 symptoms, demographic variables, and their temporal interactions
Yamamoto et al. [44]	Age: OR 1.049 (95% CI 1.024–1.077, *p* < 0.0001); Male gender: OR 1.671 (95% CI 1.075–2.613, *p* < 0.05); Hypoalbuminemia (≤ 3.5 g/dL): OR 9.755 (95% CI 5.137–20.561, *p* < 0.001); Nil per os: OR 2.708 (95% CI 1.744–4.211, *p* < 0.001); ITG (incomplete testing group): OR 4.764 (95% CI 2.323–10.275, *p* < 0.001)	Age, gender, albumin status, nil per os status, cognitive function
Zipprich et al. [45]	OR (Backward selection): Age (OR 1.05, 95% CI 1.02–1.08), Cancer (OR 2.47, 95% CI 1.21–5.02), Surgery no/yes (OR 0.35, 95% CI 0.18–0.69), ICU no/yes (OR 3.51, 95% CI 1.53–8.07), ISAR (OR 1.21, 95% CI 1.05–1.40), Chair rising test (OR 1.01, 95% CI 1.00–1.01), Polypharmacy ≥ 6 drugs/day (OR 1.99, 95% CI 1.07–3.70), Electrolyte imbalance no/yes (OR 2.35, 95% CI 1.26–4.37), Urinary catheter no/yes (OR 3.36, 95% CI 1.82–6.19), Fixation/restraints no/yes (OR 2.46, 95% CI 1.28–4.72). OR (Forward selection): Age (OR 1.04, 95% CI 1.02–1.07), Cancer (OR 2.53, 95% CI 1.25–5.12), ICU no/yes (OR 3.30, 95% CI 1.45–7.50), ISAR (OR 1.20, 95% CI 1.05–1.37), Chair rising test (OR 1.01, 95% CI 1.00–1.01), Polypharmacy ≥ 6 drugs/day (OR 1.98, 95% CI 1.07–3.66), Electrolyte imbalance no/yes (OR 2.36, 95% CI 1.27–4.40), Urinary catheter no/yes (OR 3.30, 95% CI 1.80–6.05), Fixation/restraints no/yes (OR 2.37, 95% CI 1.25–4.50). RR: Predisposing factors → Restricted mobility (RR 2.13, 95% CI 1.37–3.32), Cognitive deficits (RR 1.99, 95% CI 1.22–3.23), Higher age (RR 1.84, 95% CI 1.15–2.94), Polypharmacy ≥ 6 drugs/day (RR 1.72, 95% CI 1.07–2.76). Hospital‐acquired factors → Electrolyte imbalance (RR 1.93, 95% CI 1.20–3.09), Urinary catheter (RR 3.06, 95% CI 1.97–4.74), Fixation/restraints (RR 2.27, 95% CI 1.45–3.54), Mechanical ventilation (RR 3.15, 95% CI 1.87–5.30), ICU stay (RR 2.79, 95% CI 1.77–4.40).	Predisposing risk factors, Hospital‐acquired or trigger risk factors. The fitted Cox proportional hazards regression revealed the effects of age, presence of cancer, surgery, and stay in the ICU interactions with ward changes.

*Note: Not applicable* means that the type of information required was not feasible to obtain based on the paper's data. *Not reported* implies that, based on the paper's data, these might be calculated, but still, they were not reported.

**TABLE 4:** Long‐term outcomes.

Authors and publication year	Follow up duration	Cognitive domains	Effect size
Bertschi et al. [16]	Not applicable	FIM (Comprehension, Expression, Social interaction, Problem solving, Memory)	Not applicable
Durlach et al. [17]	At hospitalization and three months after discharge.	None (no formal cognitive testing)	Not reported
Fernández‐Gonzalo et al. [18]	1 month after ICU discharge	Premorbid intelligence quotient (IQ) estimation Verbal attention and working memory Visual attention and working memory Learning, short‐ and long‐ term verbal memory Visual memory Speed of processing Speed of processing and Executive function (Automatic response inhibition) Speed of processing and Executive function (Flexibility) Executive function (phonetic verbal fluency)	Not reported (group comparisons only)
Foran et al. [19]	Not applicable (no post‐discharge follow‐up; assessments conducted during hospitalization, including inpatient test–retest)	Global cognition, executive function (MMSE and FAB)	Not applicable
Hallgren et al. [20]	Up to 25 years	Verbal, spatial/fluid, memory, processing speed, and global cognitive composite score	Not reported (only growth model estimates provided)
Hammami et al. [21]	Not applicable (no follow‐up after discharge; data collected during hospital stay)	Global cognition (MMSE)	Not applicable
Hou et al. [22]	Not applicable (cross‐sectional study)	Global cognition (MMSE and clinical diagnosis).	Not applicable
Jiménez Mola et al. [23]	Not applicable (cross‐sectional study)	Global cognition (classified as no impairment, mild impairment, moderate/severe impairment according to DSM‐V criteria)	Not applicable for long‐term outcome (cross‐sectional study). Effect size reported as R^2^ in comparative analyses
Kamalzadeh et al. [24]	Not applicable (cross‐sectional study)	Global cognition based on orientation to time and place, registration and short‐term recall, language, concentration, and visual construction (MMSE) and memory (Mini‐Cog)	Not applicable
Karnatovskaia et al. [25]	3 months follow up (questionnaires and MoCA‐blind by telephone; Medical data was abstracted from the electronic health record)	Global cognition (MoCA‐blind)	PT in ICU predicting 3 m HADS‐D: *β* = −2.08 (95% CI −3.21, −0.96, *p* < 0.001); Depression predicting 3 m HADS‐D: *β* = 1.67 (95% CI 0.62–2.73, *p* = 0.002); Non‐invasive ventilation predicting 3 m MoCA‐Blind: *β* = 1.33 (95% CI 0.50–2.17, *p* = 0.002); In‐hospital MoCA‐Blind predicting 3 m MoCA‐Blind: *β* = 0.52 (95% CI 0.39–0.65, *p* < 0.001)
Kindstedt et al. [26]	Not applicable (cross‐sectional study).	Global Cognitive screening (Gottfries' 4‐item cognitive scale).	Not applicable.
Kunicki et al. [27]	After discharge, follow‐up assessments occurred at 1, 2, 6, 12, 18, 24, 30, 36, 48, 60, and 72 months.	Global cognition (General Cognitive Performance composite score, assessing memory, language, executive function, and attention).	Postoperative delirium associated with a slope of cognitive decline of 0.14 population SD units/year, 0.04 faster than the 0.10 SD/year in comparison groups without surgery or delirium (40% faster rate of decline).
Lagarto et al. [28]	Not applicable (no follow‐up after discharge; data collected during hospital stay)	Global cognition (MMSE)	Not applicable
Mahanna‐Gabrielli et al. [29]	Neuropsychological battery three months after surgery, at patient's home.	Composite score (Memory, language, speed, executive function)	Not applicable.
Mastaleru et al. [30]	Not applicable (cross‐sectional study)	Global cognition (MMSE)	Correlation coefficients: MMSE (r = −0.094), MNA (r = −0.151), GDS (*r* = 0.093)
Miao et al. [31]	Cognitive function was measured before discharge and at 1‐month post‐discharge.	Global cognition (Mini‐Cog)	Not reported
Mourao et al. [32]	Not applicable (no follow‐up after discharge; data collected after 72 h of hospitalization, and at hospital discharge)	Global cognition, executive function (MMSE and FAB)	Not applicable
Mudge et al. [33]	Not applicable (cross‐sectional study)	Global cognition (4AT)	Not applicable
Mutchie et al. [34]	Not applicable (cross‐sectional study)	Cognitive status (Modified Mini‐Mental State Examination 3MS)	Not applicable
Nagae et al. [35]	Not applicable	Global cognition (MMSE)	Not reported
Niu et al. [36]	Not applicable (cross‐sectional study)	Visuospatial/executive function, naming, memory, attention, language, abstraction, delayed recall, and orientation (MoCA)	Not applicable (R^2^ values reported correspond to variance explained in cross‐sectional regression models, not to longitudinal effect sizes over a follow‐up period.)
Nomura et al. [37]	1 month and 1 year post‐surgery	Composite cognitive Z‐score (global cognition)	*β*‐coefficient (Baseline to 1 month, Frail vs. Nonfrail: *β* = −0.35, 95% CI –0.69 to −0.0079, *p* = 0.045). *β*‐coefficient (Baseline to 1 year) *β* = 0.004 (95% CI –0.32 a 0.33, *p* = 0.979)
Shami et al. [38]	Not applicable (no cognitive function follow‐up. 30‐, 60‐, 90‐day readmission and 1‐year post‐discharge mortality)	Global cognition (Mini‐Cog)	1‐year survival: OR = 0.82 (95% CI: 0.53–1.27)
Sprung et al. [39]	15‐month intervals (median 3.2 years after first hospitalization)	Memory, attention/executive function, language, and visuospatial skills (global cognition)	Annual slope change in cognitive z‐scores—Global cognition: −0.051 (95% CI: −0.057 to −0.045); Memory: −0.042 (95% CI: −0.048 to −0.036); Language: −0.028 (95% CI: −0.034 to −0.022); Visuospatial: −0.019 (95% CI: −0.023 to −0.014); Attention/executive: −0.041 (95% CI: −0.048 to −0.035).
Sumida et al. [40]	Not applicable (no follow‐up after discharge; data collected at admission and discharge)	Global cognition (FIM‐Cognitive)	*β* = 0.29 (*p* = 0.018) for ln(HbA1c); *β* = −0.29 (*p* = 0.016) for AF; *β* = −0.26 (*p* = 0.034) for ln(hs‐CRP); R^2^ = 0.377
Tolley et al. [41]	Not applicable (no follow‐up after discharge; data collected from admission and discharge)	Global cognition (sMMSE, MoCA)	Continuous model: Improvement in frailty predicted by cardiac admission B = −0.319 (95% CI −0.614 to −0.023), CIRS score B = −0.059 (95% CI −0.075 to −0.043), SPPB score B = −0.046 (95% CI −0.075 to −0.017). Worsening predicted by cognitive impairment B = 0.245 (95% CI 0.075–0.414), delirium B = 0.273 (95% CI 0.082–0.463), CCI score B = 0.034 (95% CI −0.003–0.072), HADS anxiety score B = 0.022 (95% CI 0.004–0.041)
Wiegand et al. [42]	Not applicable (no follow‐up, cross‐sectional study)	Global cognition (MMSE)	Not applicable
Wilke et al. [43]	Not applicable (no follow‐up after discharge; 3 repeated assessments within one week during hospital stay)	Orientation, verbal memory, attention, language and visuospatial praxis (MMSE)	Regression estimates (*β*): MMSE +0.93/day for male sex × time; MMSE +0.81/day for prediagnosed dementia × time; DRS‐R‐98 –1.42/day
Yamamoto et al. [44]	Not applicable (cross‐sectional study)	Global cognition (STMT‐R)	Mortality: CDG vs. NCDG: OR 1.888 (95% CI 0.977–3.871, *p* = 0.059); ITG vs. NCDG: OR 4.764 (95% CI 2.323–10.275, *p* < 0.001)
Zipprich et al. [45]	6, 12, 18, and 36 months. Longitudinal outcome measures after 6 months included the Karnofsky Performance Score (KPS), the 3‐level EuroQol (EQ)‐5D‐3L, and mortality as well as the IQCODE for those patients who were assessed with the IQCODE at baseline.	Global cognition (MoCA)	Adjusted HR for all‐cause mortality after delirium: 2.2 (95% CI = 1.4–3.4); after 2‐CAM state: 1.8 (95% CI = 1.2–2.7)

*Note: Not applicable* means that the type of information required was not feasible to obtain based on the paper's data. *Not reported* implies that, based on the paper's data, these might be calculated, but still, they were not reported.

## 3 Results

From the 30 studies included in this scoping review, in terms of the study design, 10 were cross‐sectional studies, 11 were observational (some of them with prospective data analysis), one was a secondary data analysis, two were retrospective, and 6 were longitudinal studies.

In terms of sample features, 25 of the studies reportedly focused on older adults with no significant cognitive issues at the time of hospital admission; two studies had a mixed sample of older adults with normal cognitive aging and participants with cognitive decline [22, 44]; two recruited samples in different stages of frailty [29, 37] and one recruited a mixed sample of patients with and without delirium [45].

In relation to the cognitive measures used in the studies, 14 studies relied on a single screening test to characterise the cognitive status of the participants. Thus, seven studies [16, 21, 22, 30, 35, 42, 43] used only the MMSE to assess cognitive status, while another one [23] used DSM‐V criteria; one study relied only on the MoCA‐Blind [25]; another research [26] used the 4‐item version of the Gottfries' cognitive scale; two studies [31, 38] used only the Mini‐Cog; another two relied respectively on the Modified MMSE [34] and on the MoCA [36]; and another one [40] used the Functional Independence Measure (FIM‐Cognitive).

Four studies used a maximum of two screening measures, such as MMSE and FAB [19], MMSE and Mini‐Cog [24], MMSE and MoCA [41], or MMSE and STMT‐R [44].

The combination of a screening test and an informant questionnaire was only reported in three studies [18, 28, 45], while another one [27] used it in the context of a broader neuropsychological battery. The use of such a battery was only reported in Kunicki et al. [27] as well as five other studies [18, 20, 29, 37, 39].

Furthermore, the frequency of assessment showed a wide variation between studies. Thirteen of the reviewed studies did not specify the timing of assessments, stating only that they were conducted during the hospital stay. Nine studies conducted a single assessment; of these, eight were performed within the first few days of admission, while one assessed patients post‐discharge. Only six studies conducted multiple evaluations at different times [25, 27, 29, 31, 37, 40]. One study did not provide any details regarding the assessment process, while another conducted assessments every three years, unrelated to hospital stay.

With relation to the length of hospital stay, up to 14 studies did not clearly specify the length of stay for patients, including one that only specified days before surgery [19, 20, 22, 23, 24, 26, 27, 29, 30, 31, 36, 37, 42, 45]. From the remaining 16, thirteen studies showed a wide range of median lengths of hospitalization. Five of them showed less than 10 days of median stay [25, 33, 34, 38, 39], with another five reporting between 10 and 19 days of median length of stay [16, 18, 28, 32, 35], and three showing 20 or more days of median length of hospitalization [21, 40, 43]. The last three studies had mixed samples with different hospitalization stays. Durlach et al. [17] separately reported days in ICU and in the inpatient general ward, which ranged (ICU + general ward) from 3 days for non‐delirium patients, 8 for subsyndromal patients, and 9 for delirium patients. Tolley et al. [41] reported a median of 7 days for those in the acute hospitalization unit, and a median of 20 for those in geriatric rehabilitation. Finally, Yamamoto et al. [44] differentiated between those without cognitive dysfunction (median of 31 days ± 2.89 days), those with cognitive dysfunction (median of 40.42 days ± 2.21 days), and those unable to complete the test (median of 54.66 ± 3.78 days).

In terms of the prevalence of cognitive decline, ten of the reviewed studies specifically reported a high prevalence of cognitive decline among hospitalized older adults, with some of them highlighting that cognitive decline was most marked in patients with prolonged hospital stays or those with pre‐existing cognitive impairments [25, 31, 33, 34, 36, 38, 39, 41, 42, 44]. More specifically, Karnatovskaia et al. [25] registered the highest prevalence with 58% of participants experiencing cognitive decline, followed by Mudge et al. [33], who observed a rate of 43% (in 92 out of 216 patients); Shami et al. [38] with a prevalence of 35%; and Mutchie et al. [34] with one‐third of participants exhibiting cognitive decline.

Additionally, other studies provided some information related to cognitive status or decline within their samples [16, 19, 20, 22, 23, 26, 32, 35, 40, 45]. Fernández‐Gonzalo et al. [18] identified three cognitive phenotypes in their studied sample, critically ill mechanically ventilated survivors, using the unsupervized machine learning K‐means clustering algorithm. Four studies included information related to symptoms of acute cognitive dysfunction; they specifically focused on delirium [17, 27, 29, 43], while five other studies directed attention to fragility [21, 29, 30, 37, 41]. Two different studies included information related to dementia; Kamalzadeh et al. [24] reported a prevalence of 22% in their study, whereas Lagarto et al. [28] reported a prevalence of 43.5%. Both categorized their sample into two groups: demented and non‐demented.

In relation to factors contributing to cognitive decline during hospitalization, older age is frequently cited as the primary contributor, being highlighted in 17 of the studies analysed. Moreover, factors such as being a female, suffering from depressive symptoms, a lower education level, or length of hospital stay are also referred to in various studies [20, 22, 31, 35, 44]. More specifically, gender was referenced in 10 of the reviewed studies, while four of them indicated that males had worse cognitive outcomes [25, 34, 43, 44]; another two suggested that females were more affected [18, 24], and four studies showed no gender differences [23, 31, 36, 42]. Depressive symptoms were mentioned in 18 of the studies, while lower education level appeared in eight, and length of hospitalization in ten of them. Some studies further emphasized the importance of lower health status, malnutrition or dysphagia, frailty or dementia [17, 21, 24, 29, 30, 32, 37, 38, 42]. Dementia was referenced in half of the reviewed studies, whereas malnutrition and dysphagia were addressed in ten studies. Frailty received comparatively less attention, being discussed in eight studies. Hou et al. [22] also highlighted the role of central obesity, smoking, diabetes, and hypercholesterolemia as risk factors for cognitive decline, whereas Lagarto et al. [28] indicated that delirium and reduced arousal were significant predictors of cognitive decline during hospitalization. Finally, Sprung et al. [39] reported that nonelective hospitalizations and hospitalizations for medical indications were also associated with the acceleration of cognitive decline.

Finally, reviewed studies show that prolonged hospital stays were associated with greater functional decline, an increased risk of institutionalization, mortality, or rehospitalization after discharge as the main long‐term outcomes [17, 23, 24, 25, 31, 33, 37, 38, 42].

In summary, regarding the main outcomes, this scoping review has identified some key findings related to cognitive decline in hospitalized older adults: (1) heterogeneous sample characteristics, (2) diversity of cognitive measures used, (3) variability in the way to report the length of hospital stay, (4) oscillations across studies of the prevalence of cognitive decline, (5) variety of factors contributing to cognitive decline, and (6) established long‐term effects.

## 4 Discussion

The main goal of the current scoping review was to provide a clearer view of the cognitive function during hospitalization in older adult patients. In order to achieve this goal, we investigated how existing studies have examined the association of hospitalizations with cognitive decline among older inpatients. Specifically, our research focused on identifying assessment tools used, frequency of evaluations during hospitalization, types of samples collected, and length of hospital stay, as reported in previous literature.

Results from this scoping review provided information related to cognitive decline in hospitalized older adults, more specifically, in terms of prevalence of cognitive decline, factors contributing to that cognitive decline, and long‐term effects after discharge from the hospital. In relation to prevalence of cognitive decline, variability among percentages of prevalence highlights the inconsistency of cognitive decline rates across studies, populations, and methodologies [25, 31, 33, 34, 36, 38, 39, 41, 42, 44]. Even though these figures are not always conclusive, some of the high percentages observed warrant careful consideration. It is possible that the variety of cognitive screening tools used across the studies, as well as the diversity of assessment moments in which these tools were used, may affect the estimation of the prevalence of cognitive decline across reviewed studies.

Regarding different factors contributing to that cognitive decline, older age consistently emerged as a key factor. In contrast, other factors such as gender, depressive symptoms, lower education level, length of hospital stay, lower health status, malnutrition or dysphagia, frailty or dementia were less frequently reported. These results suggest that, although older age is a widely recognized factor, other influential factors may still be underexplored. Further research is needed to clarify the influence of gender in cognitive outcomes. A broad, well‐balanced sample may be crucial in providing more robust insights. Along with this, it may be valuable to explore factors such as dementia, depressive symptoms, and length of hospital stay in greater depth to better understand their role in cognitive status among older adults during hospitalization.

Lastly, concerning the long‐term effects of cognitive function in older adults during hospitalization, our scoping review identified various outcomes. Prolonged hospital stays were often associated with greater functional decline, increased risk of institutionalization, mortality, or rehospitalization after discharge.

In terms of the clinical significance and implications of the results, the findings from this review may help improve the outcomes for this population. More specifically, these findings underscore the need for appropriate cognitive screening and targeted interventions for older patients to mitigate the long‐term negative effects associated with hospital stays [17, 23, 24, 25, 31, 33, 37, 38, 42].

This review has limitations due to its own nature as a scoping review. A systematic review with meta‐analysis was not feasible due to the limited research in this area and the heterogeneity of existing studies. As previously mentioned, the available literature on the topic is limited, with some research results being highly specific while others lacking sufficient depth, making it difficult to achieve definitive conclusions.

First, a major limitation is the widespread use of screening methods. The vast majority of studies reviewed relied on different general screening tools (e.g., MMSE, MoCA, FIM‐Cognitive, or Mini‐Cog) to evaluate cognitive function, which may not capture the full extent of cognitive status. Relying solely on these screening tools, which typically provide only a general score, may result in oversimplification of the cognitive assessment. This screening approach can overlook important details, such as impairment in specific cognitive domains or error patterns consistent with particular neurodegenerative diseases. In contrast, a smaller proportion employed comprehensive neuropsychological batteries providing a more in‐depth assessment. Moreover, the variability in assessment tools affects the comparison between studies, challenging possibilities to draw consistent conclusions. Furthermore, the frequency of assessment varied widely across studies. Nearly half of the studies did not specify the timing of assessment during hospitalization. Some conducted multiple evaluations at different time points during and after hospitalization, while others assessed patients only once, either at the beginning of their hospital stay or post‐discharge. This inconsistency in assessment timing further complicates a clear vision in assessment methods.

Second, the length of hospital stays was not clearly specified in nearly half of the included studies. Additionally, a substantial number of studies reported only a wide range of median hospitalization durations. A clear and consistent report of hospitalization duration may be key to better understanding cognitive outcomes in relation to the length of stay. Furthermore, there was a lack of informant questionnaires, with most studies focusing solely on assessing the patient, thereby missing valuable insights into cognitive status that could be provided by caregivers. Finally, available studies did not provide enough information to explore further whether or how in‐hospital environmental factors (e.g., disruptions, sleep disturbances) or patient‐related factors (e.g., medication use) contributed to cognitive decline.

In summary, to our knowledge, this is the first scoping review to investigate cognitive decline in hospitalized older adults, aiming to provide a clearer understanding of how existing studies have examined the association of hospitalization with cognitive status in this population.

Future research should aim for longitudinal assessments, by means of evaluating patients at multiple time points (e.g., at hospital admission and after 2 weeks, as a minimum), which would offer a clearer way to monitor cognitive evolution and capture changes during hospitalization with greater accuracy. Including a two‐week follow‐up assessment after routine admission cognitive evaluation may aid in monitoring cognitive status in hospitalized older adults. This follow‐up should monitor preservation of cognitive function during hospital stay and also may allow for timely identification of specific cognitive decline. Additionally, the establishment of an international, common standardized assessment protocol is essential to thoroughly address key cognitive domains associated with cognitive decline during hospitalization. Incorporating caregiver input is also recommended to establish a more clear view of patients' cognitive status throughout their hospital stay. Developing an assessment protocol that includes proxy information and tests covering cognitive domains known to decline during hospitalization, as identified in previous literature, is a critical gap for future research.

## Conflicts of Interest

The authors declare no conflicts of interest.

## Data Availability

The data that support the findings of this study are available from the corresponding author upon reasonable request.
